# Advances in semiochemicals of the sweet potato weevil, *Cylas formicarius*, and its application in pest management

**DOI:** 10.1007/s44297-026-00087-2

**Published:** 2026-09-03

**Authors:** Shuyan He, Chao Li, Yulin Gao

**Affiliations:** 1 Institute of Bast Fiber Crops and Center of Southern Economic Crops, Chinese Academy of Agricultural Sciences, Changsha, China; 2 State Key Laboratory for Biology of Plant Diseases and Insect Pests, Institute of Plant Protection, Chinese Academy of Agricultural Sciences, Beijing, China

**Keywords:** Sweet potato, *Cylas formicarius*, Semiochemical, Sex pheromone, Plant volatiles, Integrated pest management

## Abstract

The sweet potato weevil (SPW), *Cylas formicarius*, is one of the most destructive pests of sweet potato, *Ipomoea batatas* L., across tropical and subtropical regions worldwide. The concealed feeding habits of larvae and the nocturnal activity of adults render conventional chemical-based control largely ineffective. Semiochemical-based management, especially the use of female-produced sex pheromone, has emerged as a promising strategy for integrated pest management (IPM) for this pest insect. This review provides a comprehensive analysis of the chemical ecology of *C. formicarius*, including the identification and behavioral activity of the sex pheromone, the diversity and ecological functions of sweet potato plant volatiles, their roles in SPW’s host finding, oviposition, and direct defense, and the development of optimized trapping systems. Applications of the sex pheromone in monitoring, mass trapping, and mating disruption are discussed, together with factors influencing trap efficacy, such as design, color, height, dose, and lure longevity. The integration of pheromone-based tactics with host plant resistance and biological control agents is also addressed. Future research directions for improving the practical use of semiochemicals against this serious pest are proposed.

## Introduction

The sweet potato weevil (SPW), *Cylas formicarius* (Coleoptera: Brentidae), is one of the most devastating pests of sweet potato, *Ipomoea batatas* L., in tropical and subtropical production areas worldwide [1–4]. Both larvae and adults of SPW cause damage to sweet potato plants, and larvae tunnel into storage roots and vines, while adults feed on foliage, vines, and roots [5]. Even low infestation levels can induce the production of bitter-tasting and toxic sesquiterpenes such as ipomeamarone in storage roots, resulting in unmarketable crops [6–8]. Yield losses due to *C. formicarius* commonly range from 60 to 97%. In China’s Guangdong Province, yield generally shrunk by 5–20% and, in some cases, by up to 80% [8]. In Vietnam, farm-level plantation losses were documented to suffer up to a 40% reduction in yield [9, 10]. Losses of 3–80% were recorded in Indonesia throughout several locations and seasons, with higher damage observed during the dry season [11]. In Malaysia, exact losses in the field were not recorded in recent years; the only documented figure was a 1970 study showing 80% yield loss or 4 tons/acre [12]. In the Philippines, sweet potato yield was reduced by 50% due to *C. formicarius* infestation [13], while in Japan (Amami Islands), losses were recorded at 15% [14–16]. In the United States, the southern states of Alabama, Louisiana, Mississippi, and North Carolina produce 75% of the sweet potato supply [17]. While the main pests affecting sweet potato plantations in these regions include sweet potato weevils, wireworms, white grubs, sweet potato flea beetle, cucumber beetle, white fringed beetle, and sugarcane beetle, weevils cause the worst damage to this crop [17]. In southern Florida, up to 80% (average 69%) of losses in sweet potato yields have been reported, mainly contributed by the death of infested plants [18]. The immature stages develop cryptically within roots or vines, and adults are predominantly nocturnal, which severely limits the effectiveness of conventional insecticide-based management approaches [19–22].

In recent years, efforts have been focused on exploring semiochemicals as tools for SPW management. Semiochemicals serve as chemical signals and cues for insects to construct information about their environment and play a key role in interactions between insects and other organisms [23–25]. The sex pheromones produced by females are specific and important species-recognition signals that attract conspecifics for mating[26]. Small changes in the pheromone release rate or ratio of the chemical constituents can affect the attraction and reproductive behavior of males [27, 28]. Therefore, sex pheromones can be used to develop effective pest control strategies, for example, monitoring pest populations, mass trapping, and mating disruption [29–31].

Semiochemicals can be employed to manage herbivore populations through multiple approaches, such as using natural or synthetic molecules as attractants or repellents [32–36]. However, the application of semiochemicals as pest management agents is influenced by multiple factors, including insect species, environmental conditions, and different biotic and abiotic stresses [37–39]. The development of synthetic semiochemicals influencing pest insect preference would be a significantly promising practice in the field of chemical ecology. Such knowledge could facilitate the development of strategies that reduce conventional insecticide dependency through targeted pest control, offering greater alternatives and minimal negative impacts on beneficial organisms, ultimately constructing sustainable and eco-friendly pest management systems in agricultural fields. This review provides comprehensive knowledge and analysis of the literature on the semiochemicals of SPW as well as their existing strengths, limitations, and prospects to aid in the future application of semiochemicals in pest management.

## Sex Pheromone of *C. formicarius*

### Identification and chemical structure

The sex pheromone of *C. formicarius* was first identified from volatiles collected from virgin females and characterized as (Z)−3-dodecen-1-ol (E)−2-butenoate [40]. This crotonate ester represented the first example of a pheromone containing a butenoate moiety in insects. Later studies on feral populations from Cuba confirmed the same compound as the sole active component, with females emitting approximately 20 pg of pheromone per day [41]. The synthetic pheromone was shown to be qualitatively and quantitatively indistinguishable from the natural product in both laboratory bioassays and field trapping experiments [16].

### Electrophysiological and behavioral activity

Electroantennogram (EAG) studies demonstrated that male SPW antennae respond to the synthetic pheromone in a dose-dependent manner, whereas female antennae show no detectable response, indicating the sex-specific nature of pheromone perception [41]. The terminal crotonate function proved critical for electrophysiological activity, as the formate, acetate, propionate, and butyrate analogues of (Z)−3-dodecenol failed to elicit EAG responses from male antennae [41]. Scanning electron microscopy revealed several types of antennal sensilla, with sensilla trichoidea type 1 (long hairs, 100–150 μm) being much more abundant in males than in females, suggesting their likely role in pheromone detection [41].

In behavioral experiments using a dual-choice olfactometer, male weevils displayed maximum attraction to pheromone doses between 50 and 1000 ng, with activity peaking between the fourth and eighth hour of the scotophase [41]. The stereochemical purity of the pheromone can influence field capture effectiveness; formulations containing ≥ 94% of the (Z, E)-isomer were highly attractive, whereas the (Z, Z)-isomer at up to 5% did not show synergistic or inhibitory effects, and the alcohol precursor was inactive [41, 42].

### Perception of pheromone in C. formicarius

Recent advances in molecular biology have begun to elucidate the olfactory mechanisms underlying pheromone perception in *C. formicarius*. Three odorant binding protein genes (CforOBP1-3) were cloned and characterized. Quantitative real-time PCR assays revealed that CforOBP1 was highly expressed in the antennae and legs of both sexes, whereas CforOBP2 and CforOBP3 were predominantly expressed in the antennae of males [43]. Fluorescence competitive binding assays demonstrated that all three CforOBPs exhibited strong binding affinities to the sex pheromone ligand (Z)−3-dodecenyl-(E)−2-butenoate and to selected host plant volatiles. RNA interference-mediated knockdown of CforOBP1-3 resulted in partial anosmia, where RNAi-treated SPW showed a reduced ability to respond to both sex pheromones and host plant volatiles. These functional analyses suggest that CforOBP2 and CforOBP3 are primarily involved in male mating behavior, whereas CforOBP1 participates broadly in host and mate finding [43]. Understanding the molecular mechanisms of SPW pheromone perception could facilitate the development of novel interferents targeting olfactory pathways.

### Pheromone dose and lure longevity

Field studies on Guam compared doses of 10 μg and 100 μg of pheromone loaded onto rubber septa and found that the 100 μg dose captured significantly more adults [16]. Lure longevity trials indicated that septa remained capable of capturing weevils for up to 98 days, although capture rates declined progressively with age. Traps baited with septa aged 0–28 days caught the most weevils, and a replacement interval of approximately 30 days was recommended for maximum attraction [16].

## Sweet potato volatile organic compounds and their roles

### Diversity of sweet potato volatiles

Sweet potato plants release a complex blend of volatile organic compounds (VOCs), the composition and quantity of which vary considerably among cultivars, tissue types, and physiological states [44–47]. These compounds may act as attractants or repellants for different herbivores, as illustrated in Table 1.

**Table 1:** SPW behaviors towards sweet potato plants’ volatile chemicals

Source	Volatile compounds	Activity	Reference
Root	(E, E)−2,4-heptadienal; (E, E)−2,4-decadienal; E- β-ionone; F- β-ionone; G- geranylacetone	Attractive	Liu et al., 2024 [48]
Leaf	(Z) −3-hexenyl acetate; *allo*-ocimene	Repellent	Xiao et al., 2023 [49]
Root	Nerol; Z-citral; Methyl geranate; E-citral	Attractive	Wang et al., 2002 [50]
Root	α-gurjunene; α-humulene; Ylangene	Repellent	Wang et al., 2002 [50]
Root	Linalool; Citronellol; Nerol; Geraniol	Repellent	Liu et al., 2023 [51]
Root	Eucalyptol; β-carotene; Benzaldehyde; Vanillin; Phenethyl alcohol	Attractive	Wang et al., 2021 [52]
Flower	2-(2-butylcyclopropyl)- cyclopropanenonanoic acid methyl ester	Repellent	Korada et al., 2010 [53]
Root	9,12-(Z, Z)-octadecadienoic acid	Repellent	Korada et al., 2010 [53]

Early work by Horvat et al. identified 21 volatile compounds across three sweet potato cultivars, including aldehydes, ketones, furans, pyridine, alcohols, terpenes, and palmitic acid [54]. A recent comprehensive analysis of 40 sweet potato varieties with different flesh colors detected a total of 121 volatile compounds by headspace solid-phase microextraction coupled with gas chromatography–mass spectrometry (HS-SPME/GC–MS), with aldehydes, furans, and terpenes being the most abundant chemical classes [48]. Among these, 35 compounds were identified as key aromatic compounds (odor activity value > 1), of which 17 were found in all varieties and thus considered the major components contributing to sweet potato aroma. Notably, yellow-fleshed varieties exhibited the strongest overall aroma, attributed largely to aldehydes such as (E, E)−2,4-heptadienal and (E, E)−2,4-decadienal derived from unsaturated fatty acid degradation. Orange-fleshed varieties were characterized by apocarotenoid volatiles, including E-β-ionone, β-ionone, and geranylacetone, whereas purple-fleshed varieties displayed weak aromas but significantly elevated terpenoid concentrations. A study on sweet potato shoots identified 46 volatile compounds when they were invaded by sweet potato weevils. Among these volatiles, (Z)−3-hexenyl acetate (z3HAC) and *allo*-ocimene were released at high levels, and both volatiles exhibited a negative effect on the orientation of SPW to sweet potato plants [49]. This extensive chemical diversity provides the olfactory basics against which insect-plant interactions occur.

### Volatile-mediated host location and attraction

Volatile organic compounds emitted by sweet potato plants play a critical role in host location by *C. formicarius*. Nottingham et al. first demonstrated, using a dual-choice olfactometer, that both male and female weevils were attracted to volatiles from sweet potato leaves and a methylene chloride leaf extract [55]. Females, but not males, responded to volatiles from storage roots and root extracts, indicating sex-specific responses to different plant parts. Leaves and storage roots from four cultivars (Centennial, Jewel, Resisto, and Regal) were all attractive to female weevils; however, the attractant response varied significantly among cultivars, suggesting genetically determined differences in volatile profiles. GC and GC–MS analyses identified the major volatile constituents from Jewel leaves as sesquiterpenes [55].

Subsequently, Wang and Kays conducted detailed headspace collections and GC–MS identifications from both storage roots and aerial plant parts. In total, 33 compounds were identified, with 23 being terpenes. Three oxygenated monoterpenes, nerol, Z-citral, and methyl geranate, were found in storage root volatiles but were not detected from aerial plant parts. In olfactometer bioassays, these three compounds were demonstrated to be attractants for female weevils, with maximal attraction occurring at concentrations ranging from 0.01 to 10 nL per 10 μL hexane, and substantially higher or lower concentrations elicited reduced responses, indicating a narrow optimal range for behavioral activity. Notably, the geometric isomers geraniol and E-citral were attractive to female weevils but to a considerably lesser degree, revealing a relatively high level of binding site specificity for monoterpene perception in this insect. Interestingly, three sesquiterpenes, α-gurjunene, α-humulene, and ylangene, consistently acted as repellents at concentrations comparable to those naturally emitted by storage roots, demonstrating that sweet potato plants simultaneously emit both attractant and repellent volatiles [50]. Subsequent electrophysiological and behavioral studies further showed that VOCs from sweet potato flowers and storage roots were particularly attractive to female weevils, eliciting significant EAG responses, whereas leaf VOCs attracted predominantly males, suggesting that tissue-specific volatile blends guide distinct behavioral decisions during the host-finding process [53].

Using Y-tube olfactometers, studies demonstrated that adult weevils showed the strongest preference for storage roots (selection rate exceeding 55%), while leaves were the least preferred (selection rate approximately 30%), with no significant difference between sexes [56]. In a more recent study screening 14 sweet potato cultivars, significant differences in the behavioral responses of *C. formicarius* to different cultivars were observed, and the degree of attractiveness was correlated with cultivar resistance levels, confirming that olfactory cues are integral to herbivore resistance [57].

### Intraspecific chemical signals: larval-induced volatiles deter conspecific adults

A recent discovery unveiled the role of volatiles in mediating intraspecific competition among *C. formicarius*. Liu et al. found that sweet potato roots infested by third-instar larvae emitted a distinct volatile profile. Using GC-EAD and GC–MS analyses, five compounds eliciting consistent antennal responses from both male and female adult weevils were identified: linalool, citronellol, nerol, geraniol, and the furanoterpenoid ipomeamarone. Behavioral bioassays demonstrated that the four monoterpene alcohols significantly repelled conspecific adults from feeding and oviposition at ecologically relevant doses, with geraniol displaying the strongest repellent activity [51]. This chemically-mediated avoidance behavior is interpreted as an adaptive strategy that reduces intraspecific competition: larvae already occupying a host root signal their presence to conspecific adults via altered volatile emissions, thereby discouraging additional colonization and enhancing the survival of offspring. From an applied perspective, these monoterpene alcohols represent promising candidates for development as repellents or oviposition deterrents for SPW management [51].

### Chemosensory proteins involved in host volatile perception

**Fig. 1:**
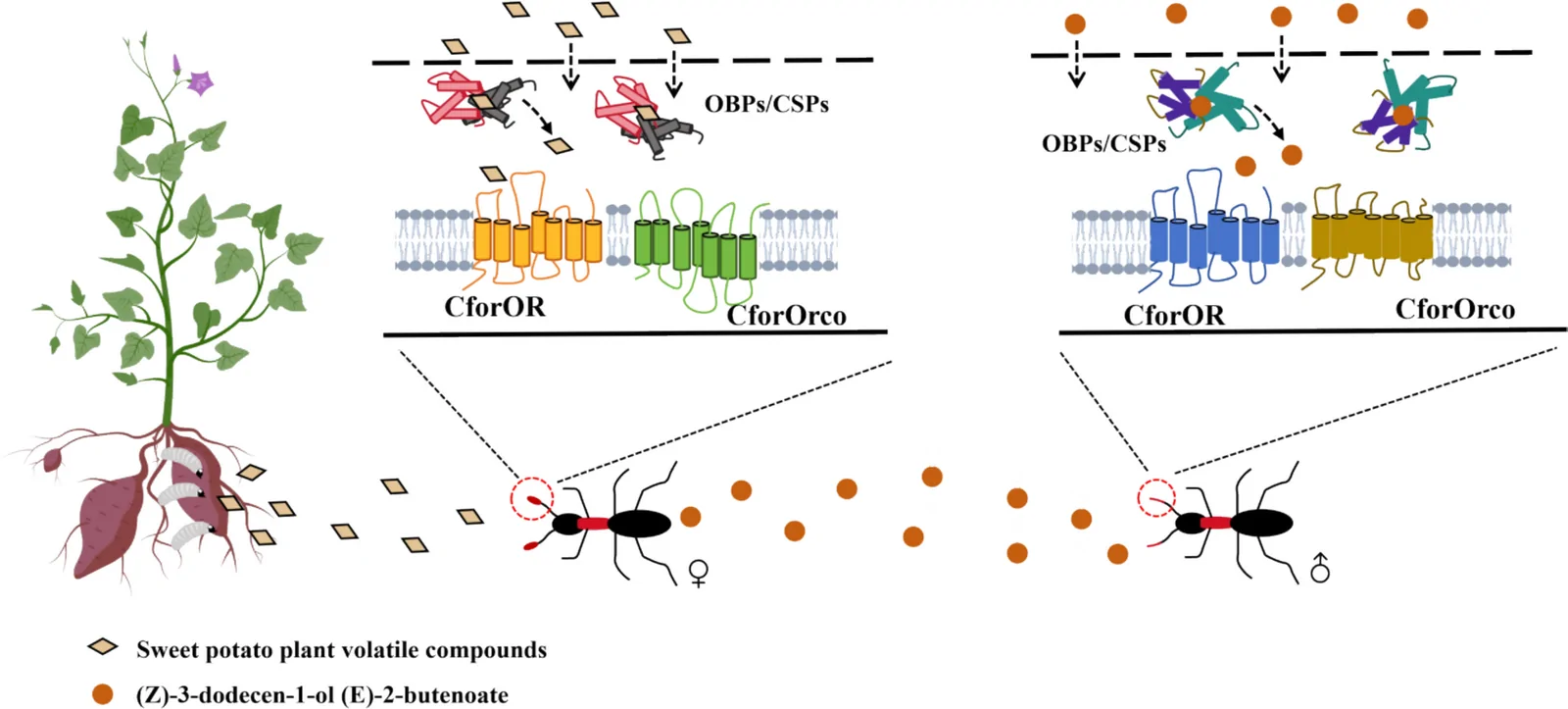
Olfactory detection mechanisms for host volatiles and sex pheromones in *C. formicarius*. Female SPW through the OBPs or CSPs to perceive sweet potato volatiles and use this chemical cue for locating host plants, male SPW recognizes the sex pheromone released by female by OBPs or CSPs and makes copulation. Abbreviations: OBPs, Odorant-Binding Proteins; CSPs, Chemosensory Proteins

Complementing the molecular insight into pheromone binding proteins, recent research has characterized the chemosensory proteins (CSPs) of *C. formicarius* that mediate the perception of host plant volatiles. Three CSP genes, CforCSP1, CforCSP5, and CforCSP6, were found to be highly expressed in the antennae of both female and male weevils. Fluorescence competitive binding assays revealed that these three CforCSP proteins exhibited high binding affinities for 17 plant volatiles, including eight host plant volatiles. Olfactometer bioassays further showed that adult weevils were significantly attracted to eucalyptol, β-carotene, benzaldehyde, vanillin, and phenethyl alcohol, whereas β-ionone elicited a repellent response. RNAi-mediated silencing of the three CforCSP genes rendered treated weevils partially anosmic to several ligands, including β-cyclocitral and benzaldehyde, and diminished their ability to locate host plant volatiles [52]. These findings elucidate the molecular interaction between host plant chemistry and weevil behavior and provide potential molecular targets for the development of novel pest management approaches (Fig. 1).

### Volatile-mediated host plant resistance

The field of volatile-mediated resistance against *Cylas* spp. has been substantially advanced beyond the sesquiterpene repellents identified by Wang and Kays [50]. Anyanga et al. investigated the chemical basis of resistance in seven field-resistant and three susceptible sweet potato varieties against the African SPW *Cylas puncticollis* and *C. brunneus*. Resistance was shown to be active rather than a means of escape. Field-resistance varieties had lower root damage and oviposition rates. This effect remains even in laboratory conditions where the SPW population was high. LC–MS analyses of root surface and epidermal extracts revealed that the concentrations of hexadecyl, heptadecyl, and octadecyl esters of caffeic acid and coumaric acid were significantly higher in resistant varieties, and these compounds were positively correlated with resistance levels. When the synthetic compounds octadecyl coumarate and octadecyl caffeate were applied to the surface of susceptible varieties, feeding and oviposition by weevils were reduced to levels comparable to those observed on resistant roots, confirming the functional role of these hydroxycinnamic acid esters in herbivore resistance [58].

In parallel work focused specifically on *C. formicarius*, Korada et al. profiled the headspace volatiles from intact plants, flowers, and storage roots across multiple sweet potato genotypes. The genotypes “Howrah, “SB IB 400/22”, and “BX 86” were least preferred by weevils in behavioral assays, whereas “Kishan” was the most preferred. GC–MS analyses identified a compound, 2-(2-butylcyclopropyl)-

cyclopropanenonanoic acid methyl ester, present in the flower headspace of the resistant genotype “Howrah”, which eluted as 9,12-(Z, Z)-octadecadienoic acid from the storage root periderm. This fatty acid is a precursor for the biosynthesis of multiple short-chain aromatic compounds through the lipoxygenase (LOX) pathway and was absent in the susceptible genotype “Kishan”. The authors proposed these cyclopropane fatty acid esters as diagnostic chemical markers for identifying sweet potato weevil resistance in breeding programs, providing a potentially powerful tool for marker-assisted selection of resistant germplasm [53].

Collectively, these studies demonstrate that chemically mediated resistance to *Cylas* weevils in sweet potato operates through multiple distinct classes of compounds, including sesquiterpene repellents, monoterpene attractants, hydroxycinnamic acid esters on root surfaces, and volatile fatty acid esters, each contributing to different aspects of herbivore defense and offering multiple targets for resistance breeding.

## Application of semiochemicals in pest management

### Monitoring

Sex pheromone-baited traps are widely used for monitoring *C. formicarius* populations [4, 59, 60]. Trap captures provide information on population density, seasonal phenology, and the need for control interventions [61]. Given that the pheromone attracts only males, monitoring programs rely on the relationship between male trap catches and subsequent larval damage [28]. Pheromone traps are particularly valuable for early detection at low population densities and for tracking the efficacy of management strategies.

### Mass trapping

Mass trapping with sex pheromone has been successfully employed to suppress *C. formicarius* populations. In a pioneering study in Okinawa, Japan, ten funnel traps deployed in a 1,200 m^2^ sweet potato field captured over 65,000 males during a 17-month period, resulting in a markedly female-biased sex ratio (approximately 80% female in the treated field vs. 50% in the untreated control), reduced mating rates among females, and substantial population suppression [62]. The male population density in the pheromone-treated field decreased to approximately one-tenth of the initial density within three months of trapping. The effectiveness of mass trapping was subsequently confirmed in other regions. In Guam, mass trapping with pheromone-baited unitraps (100 μg loading) in sweet potato fields reduced root damage to < 1 feeding hole per root compared with up to 38 holes per root in untreated controls, and yields were almost doubled, from 8.26 and 7.86 T/ha in control fields to 14.59 and 13.47 T/ha in treated fields [11]. These results demonstrate that pheromone-based mass trapping can provide significant crop protection while minimizing insecticide inputs.

### Optimization of trap design and deployment

The performance of pheromone traps is strongly influenced by trap characteristics. Comparative studies in Guam showed that Pherocon unitraps (bucket traps) captured significantly more *C. formicarius* adults than ground, funnel-water, or delta traps [4]. Medium-sized traps (13 × 17.5 cm) were more effective than larger or smaller traps. Among the tested colors, light-red traps caught the highest numbers of weevils, and the combination of a light-red trap with pheromone lure captured significantly more adults than identical traps without lures, indicating that visual and olfactory cues together govern trap efficacy. Traps placed 50 cm above the crop canopy achieved maximum captures, reflecting the tendency of adult weevils to move toward the upper parts of plants at night [4].

Subsequent work in Malaysia developed a low-cost plastic pole trap fabricated from recycled polyethylene terephthalate. Among the four window configurations tested, traps with four window openings captured 57%−72% more weevils than those with one or two windows. The detergent solution as a killing agent outperformed carbofuran and plain water. The optimized plastic pole trap, positioned 0–40 cm above the crop canopy, captured 60%−78% more weevils than commercial delta traps, wing traps, or unitraps. Interestingly, trap color did not significantly affect captures in the Malaysian system, in contrast to the Guam findings, possibly reflecting regional differences in weevil biotypes or ambient ultraviolet radiation conditions [62]. The effective attraction radius of pheromone-baited traps was estimated at 60–80 m, with a trap spacing of approximately 60 m recommended for mass trapping programs [16].

### Integration with other control tactics: synergistic enhancement

Efforts to improve the efficacy of pheromone trapping through synergistic stimuli have yielded promising results. McQuate demonstrated that green light significantly enhanced male sweet potato weevils’ response to sex pheromone, achieving up to a fivefold increase in trap captures when green light was combined with pheromone lures compared with pheromone alone [63]. This finding highlights the potential for multimodal (olfactory plus visual) lure technologies to increase trapping efficiency. The integration of pheromone trapping with biological control agents also shows considerable promise. Research on *Spodoptera frugiperda* and *Tuta absoluta* has demonstrated that pheromone lures can be combined with entomopathogenic fungi such as *Beauveria bassiana* and *Metarhizium anisopliae* in “attract-and-infect” strategies [64]. Although such combinations have not yet been fully validated for *C. formicarius*, the approach is promising and warrants further investigation. Furthermore, emerging knowledge of larval-induced repellent monoterpene alcohols [51] and of individual volatiles acting as attractants (eucalyptol, benzaldehyde, vanillin) or repellents (β-ionone) for adult weevils [52] provides a rich chemical toolbox for developing push–pull strategies tailored to *C. formicarius* in sweet potato agroecosystems.

### Host plant resistance based on volatile chemistry

Host plant resistance based on differential volatile emissions has been explored as a long-term, sustainable strategy for weevil management. The inverse correlation between sesquiterpene emission and female weevil attraction [50], together with the identification of root-surface hydroxycinnamic acid esters that confer resistance [58] and the discovery of cyclopropane fatty acid esters as diagnostic markers for resistance [53], provides breeders with multiple chemical targets for selection. The cultivar-dependent specificity of DMNT-mediated defense induction further suggests that selection for enhanced emission of defense-priming volatiles could be integrated into breeding programs [65]. Approaches combining host plant resistance with pheromone trapping are likely to yield the most robust and durable weevil management outcomes.

**Fig. 2:**
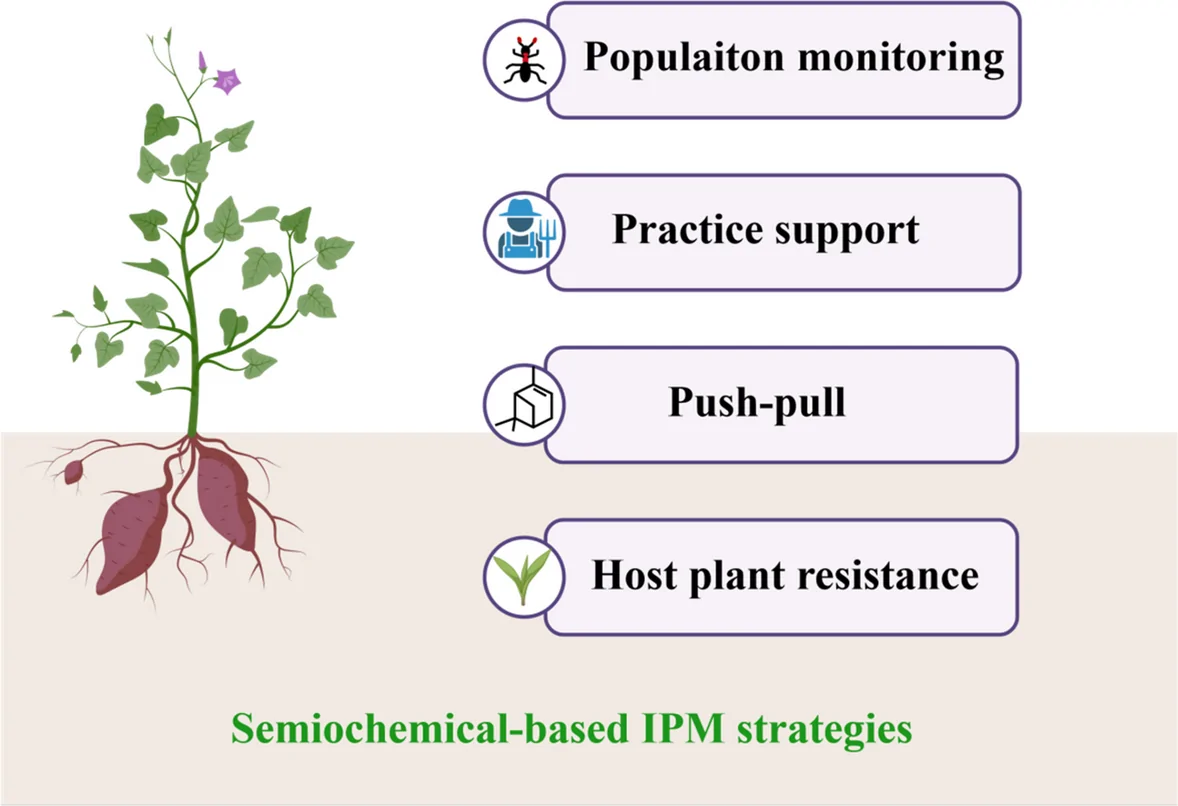
A simplified conceptual diagram of the integrated semiochemical-based IPM strategy of SPW. Female‑produced sex pheromone serves as the primary tool for monitoring, and agricultural practice decisions (e.g., mating disruption) are based on the SPW population. Host plant volatiles can be combined with pheromone to develop bisexual lures to formulat as repellent dispensers in a push‑pull strategy, and specific compounds serve as chemical markers for breeding programs

Based on the comprehensive review of semiochemicals in *C. formicarius*, a multi‑layered conceptual framework can be proposed that integrates sex pheromone, host plant attractants, larval‑induced repellents, and constitutive defense compounds into a unified pest management model (Fig. 2). At the core, the female‑produced sex pheromone serves as the primary tool for monitoring and mass trapping, with optimized trap design (light‑red color, 50 cm above canopy, 60‑m spacing) and 100 μg lures replaced every 30 days to drastically reduce male populations and disrupt mating, as demonstrated in Guam and Okinawa. The second layer exploits host plant volatiles: specific monoterpenes (nerol, Z‑citral, methyl geranate) attract both sexes, especially females, and can be combined with pheromones to develop bisexual lures, while certain sesquiterpenes (α‑humulene, ylangene) act as repellents, offering a “pull” component for trap enhancement. The third layer uses larval‑induced monoterpene alcohols (linalool, citronellol, nerol, geraniol), which deter conspecific adults from feeding and oviposition on already‑infested roots, providing a natural “push” signal to reduce intraspecific competition; these compounds can be formulated as repellent dispensers in a push‑pull strategy. The fourth layer capitalizes on root‑surface hydroxycinnamic acid esters (octadecyl coumarate and caffeate) and cyclopropane fatty acid esters, which are positively correlated with field resistance and can serve as chemical markers for breeding programs or as direct protectants applied to susceptible varieties. Finally, these olfactory tactics can be integrated with biological control agents in attract and infect systems and with cultural practices to form a resilient IPM package. The framework operates hierarchically: monitoring guides intervention timing; mass trapping suppresses male abundance; repellents (larval‑induced and sesquiterpenes) protect roots from oviposition; host resistance compounds reduce damage; and multimodal lures (pheromone + plant volatiles + green light) enhance overall efficacy. This integrated model, tailored to local conditions and pest densities, minimizes insecticide reliance while sustaining crop yield and provides a blueprint for applying chemical ecology principles to sweet potato weevil management worldwide.

## Problems and future perspectives

Despite the demonstrated efficacy of pheromone-based tactics for *C. formicarius*, several challenges remain. Mass trapping is most effective when populations are relatively low and when the immigration of mated females can be minimized. As Yasuda noted, the effectiveness of pheromone traps decreases when the crop canopy fully covers the ground, as dense foliage may prevent males within the canopy from being exposed to the pheromone plume that flows above it [62]. Variation in trap performance among geographic regions, due to differences in weevil behavior, crop phenology, or environmental conditions, underscores the need for local optimization of trap specifications [66]. The cost and availability of synthetic pheromone and traps can limit adoption by smallholder farmers in developing countries. Furthermore, the exclusive attraction of males by current lures is a limitation, and the development of bisexual lures that also attract females, perhaps by combining the sex pheromone with host plant attractants, could improve efficacy [67].

Moreover, pheromones play a fundamental role in the life history of pest insects, mediating critical behaviors such as mate location, host plant selection, and predator avoidance [68]. However, the efficacy of pheromone-based pest management strategies, including mating disruption and lure-kill, raises an important question: can herbivores develop behavioral resistance or habituation to these chemical signals?

Habituation, a form of non-associative learning, is defined as a reduced behavioral response following repeated or continuous exposure to a stimulus [69]. This phenomenon has been well documented in certain studies. For instance, male moths often show a reduced sexual response after pre-exposure to female sex pheromone [70], and continuous exposure to alarm pheromone can lead to habituation in aphid populations, where previously repellent signals no longer trigger avoidance behavior [71]. A previous study reported that habituation was achieved in pea aphids (*Acyrthosiphon pisum*) after 24-h exposure to their alarm pheromone, (E)-β-farnesene [68].

However, the occurrence of habituation appears to be highly context-dependent and varies across insect orders. Research has demonstrated that while habituation to sex pheromones is common in Lepidoptera (moths), it may not be usual in other groups, such as Homoptera, Diptera, and Hymenoptera [70]. Furthermore, stimulus intensity plays a critical role: weak stimuli tend to induce habituation, whereas strong stimuli do not.

From an evolutionary perspective, radical alterations in pheromone response could be disadvantageous, as these signals are crucial for survival and reproduction [72]. Instead of genetic resistance, habituation often represents a reversible phenotypic change, as seen in aphids, where habituated individuals can revert to sensitivity within three generations [73]. Generally, while behavioral resistance or habituation to pheromones is a genuine possibility, it is not a universal outcome. Its occurrence depends on the insect species, the specific pheromone system, and the conditions of exposure. Understanding these nuances is essential for designing robust and sustainable pheromone-based pest management strategies.

Integrating recently identified larval-induced repellents (such as geraniol) into push–pull strategies and leveraging host plant volatiles such as monoterpene attractants and/or repellent sesquiterpenes identified from storage roots represent another promising alternative.

Future research should focus on several areas. First, the molecular basis of pheromone and host volatile perception in *C. formicarius*, including the functional roles of odorant-binding proteins (CforOBPs) and chemosensory proteins (CforCSPs), which are increasingly well characterized, and these molecular targets could enable the development of novel behavioral interferant or highly sensitive biosensors for weevil detection. Second, the integration of pheromone trapping with cultural methods, host plant resistance (including the use of volatile chemical markers for screening resistant germplasm), and biological control agents should be evaluated under realistic farming conditions to develop robust IPM packages. Third, the potential of DMNT and other defense priming volatiles for enhancing crop resistance against weevil attack deserves further study, particularly to determine whether DMNT-mediated sporamin induction is effective specifically against *C. formicarius*. Fourth, simple, low-cost trapping systems that can be produced locally, as demonstrated in Malaysia, should be promoted in resource-limited regions. Finally, the development of multi-modal trapping technologies combining pheromones, plant volatiles, and visual cues such as colored traps or green light could substantially improve capture efficacy and should be systematically investigated.

In summary, semiochemical research on *C. formicarius*, encompassing the sex pheromone, an increasingly detailed understanding of host plant volatile chemistry and its behavioral and defensive functions, and the molecular mechanisms of olfactory perception, has yielded practical tools and fundamental knowledge that can reduce dependence on synthetic insecticides while protecting sweet potato yields. Continued progress in chemical ecology and integrated pest management will further enhance the sustainability of sweet potato production worldwide.

## Data Availability

Data sharing is not applicable to this article, as no datasets were generated or analyzed during the current study. Instead, it comprehensively surveys and analyzes literature, studies and published works in the field. All information, data and references cited in this review paper are duly acknowledged and properly cited within the text and in the reference list.
